# A Complicated Course of a Coronal Shear Fracture Type IV of the Distal Part of Humerus Resulting in Resurfacing Radiocapitellar Joint Replacement

**DOI:** 10.2174/1874325001711010248

**Published:** 2017-03-31

**Authors:** Ingo Schmidt

**Affiliations:** SRH Poliklinik Gera Gmbh, Straße des Friedens 122, Gera 07548, Germany

**Keywords:** Coronal shear fracture, Elbow, Headless compression screw, Posttraumatic osteoarthritis, Radiocapitellar joint, Unicompartmental resurfacing joint arthroplasty

## Abstract

**Background:**

Coronal shear fracture type IV of the distal part of humerus is a very rare injury with articular complexity potentially leading to posttraumatic osteoarthritis. One option for surgical treatment of advanced unicompartmental radiocapitellar osteoarthritis is resurfacing radiocapitellar joint replacement.

**Method:**

A 62-year- old female sustained a coronal shear fracture type IV of the distal part of left humerus that was primarily treated with open reduction and internal fixation using headless compression screws. Three years postoperatively, there was a migration of one screw into radiocapitellar joint that led to circular deep cartilage defect of radial head. Four years after ORIF, a distinctive radiocapitellar osteoarthritis has evolved leading to a resurfacing radiocapitellar joint replacement using the Lateral Resurfacing Elbow^TM^ (LRE) system.

**Result:**

At the 2-year follow-up after that procedure, there was an excellent subjective and functional outcome. Radiographically, no loosening or subsidence of implant without any signs of overstuffing could be found. The patient reported that she would have the same procedure again.

**Conclusion:**

The goal of unicompartmental radiocapitellar replacement is to obtain stability in elbow joint by avoiding cubitus valgus with subsequent instability of the distal radioulnar joint, and it does not alter the unaffected ulnohumeral joint. Additionally, the feature of the LRE^TM^ system is that the radial head is not excised, and so will receive the anatomical length of the overall radius articulating with the capitellum by preserving the annular ligament. In the literature only three publications could be found in which short-term results with the use of the LRE^TM^ system have been described. Hence, further studies are needed to validate this concept.

## INTRODUCTION

Coronal shear fractures (CSFs) of the distal part of humerus involving the capitellum and/or trochlea are rare injuries with articular complexity, and account for less than 1% of elbow fractures [1, 2]. Bryan and Morrey [3] classified capitellar fractures into four types, and McKee *et al.* [4] later modified type IV. Type I (*i.e.* Hahn-Steinthal fracture) is complete capitellar fracture with little or no extension into the lateral trochlea typically associated with anterior displacement, type II (*i.e.* Kocher-Lorenz fracture) is minimal osteochondral fracture typically associated with posterior displacement, type III (*i.e.* Broberg-Morrey / Grantham fracture) is comminuted/compression capitellar fracture, and type IV (McKee's modification of type I) is a shear fracture of the distal part of humerus that extends in the coronal plane across the capitellum to include most of the lateral trochlea ridge and the lateral half of the trochlea, identified by the presence of the pathognomonic “double arc sign” on lateral radiograph.

Complex fracture of the distal part of humerus represents a challenging therapeutic problem. It can lead to mild to moderate posttraumatic and/or postoperative osteoarthritis (OA) in up to 74% of cases at an average of 19 years, and in 40% of all cases subsequent procedures are necessary, however, clinical symptoms does not often correlate with the extend of radiographic signs [5]. The Lateral Resurfacing Elbow**^TM^** (LRE**^TM^**, Biomet, Warsaw , Indiana/USA) arthroplasty is one system that is currently in use [6-8]. We present one complicated course of the very rare CSF type IV resulting in posttraumatic unicompartmental radiocapitellar OA that was treated with this system, and a technical note of implant and short review of literature will highlight this relatively new procedure.

## TECHNICAL NOTE LRE^TM^ SYSTEM

**Fig. (1):**
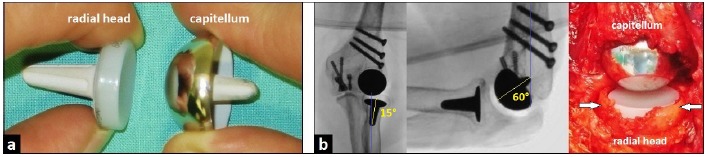
(Technical Note LRETM system): (a) Photograph demonstrating the metal-on-UHMW PE articulation. **(b)** PA an lateral fluoroscopy intraoperatively showing correct insertion of radial and capitellar components in direction of their axes, and intraoperative clinical photograph showing the cortical frame around the offset of radial UHMW PE component (arrows).

The uncemented LRE**^TM^** system is composed of two monoblock components (Fig. **1a**) and it was created and first described in 2007 by Pooley [6]. The system is available in 4 sizes (small / medium / large / extra-large). The spherical capitellar component is manufactured from cobalt-chrome (CoCr) alloy; and it has an extended skirted rim and tapered, cruciform, hydroxyapatite (HA)-coated peg to ensure secure press-fit fixation. The peg is to be inserted in direction of anatomic capitellar axis which is usually angled 50-60° anterior to the axis of the humeral shaft (Fig. **1b**). When isolated capitellar OA is present, it can be used as unicompartmental capitellar hemiarthroplasty, and there is an option for use in a cemented manner if the bone stock is poor. The radial head component has a tapered, HA-coated, cruciform CoCr stem connected to a metal-backed ultra-high molecular weight (UHMW) polyethylene (PE) articulation that provides a cortical frame around the UHMW PE offset. The peg is to be inserted in the direction of anatomic radial neck axis which is usually angled 15° lateral to the axis of the radial shaft (Fig. **1b**).

## CASE PRESENTATION

**Fig. (2):**
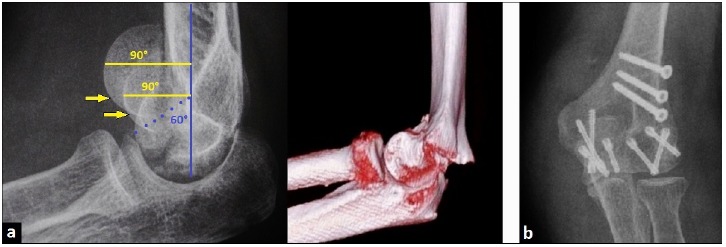
**(Case Presentation): (a)** Lateral radiograph and 3D CT scan demonstrating CSF type IV with anterior displacement of trochlea as well as capitellum of 30° (lines, points), note the pathognomonic "double arc sign" (arrows). (b) PA radiograph after primary ORIF showing correct alignment of the distal part of humerus without step-off in both articular surfaces.

A 62-year-old female presented with a CSF type IV of the distal part of left humerus after a fall on her left outstreched arm, in which his left hand took the force of the impact on the ground. On examination, there was swelling and distinctive pain-related decrease of motion in her left elbow. The diagnosis was confirmed radiographically and with the use of three-dimensional (3D) computed tomography (CT) scans. The lateral radiograph showed anterior displacement of the trochlea as well as the capitellum of 30° to normal that was associated with the “double arc sign” (Fig. **2a**). The open reduction and internal fixation (ORIF) through a dorsal incision was detected and the elbow joint was exposed through an oblique osteotomy of the lateral column of distal part of humerus. The trochlea as well the capitellum were anatomically reducted using seven 2,4 mm headless compression screws in antegrade as well as retrograde manner, and the osteotomy of the lateral column of distal part of humerus was anatomically reducted using three 3,5 mm cortical compression screws (Fig. **2b**). After surgery, the left arm was immobilized in a plaster splint for two weeks. Then, the elbow's motion was freed, and strenghtening was started after the sixth postoperative week.

**Fig. (3):**
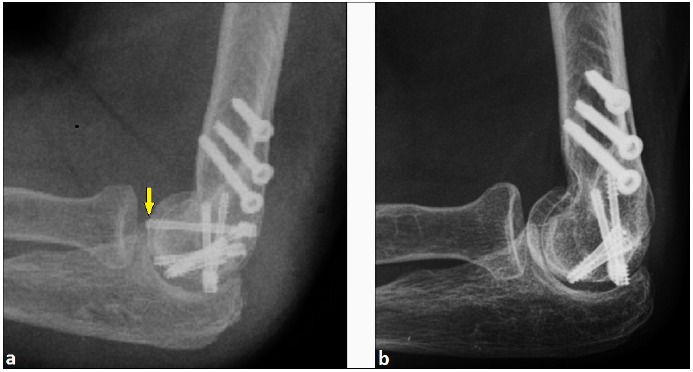
**(Case Presentation): (a)** Lateral radiograph three years after ORIF showing migration of one of the headless compression screws into radiocapitellar joint (arrow) despite complete union of CSF. **(b)** Lateral radiograph after removal of all three headless compression screws of capitellum showing no signs of posttraumatic/postoperative OA.

The further course was uncomplicated. Three years later, the patient reported increasing pain during supination and pronation of the left forearm that was clinically associated with crepitus in the radiocapitellar joint, and an elbow's extension deficit with 15° compared to the unaffected right elbow. The lateral radiograph showed intraarticular migration of one headless compression screw despite complete union of CSF (Fig. **3a**). All three screws primarily inserted into capitellum were removed (Fig. **3b**), and the patient was pain free for another year.

**Fig. (4):**
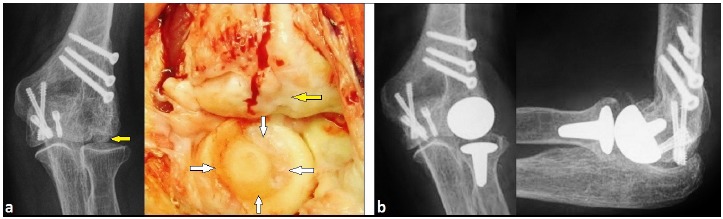
**(Case Presentation): (a)** PA radiograph and intraoperative clinical photograph four years after ORIF including one year after partial removal of headless compression screws showing distinctive OA of the capitellum (yellow arrows) without any signs of degenerative changes of radial head radiographically, but intraoperatively there was pronounced deep circular cartilage defect (white arrows). **(b)** PA and lateral radiographs showing correct positioning and alignment of LRE™ system without any signs of overstuffing.

 One year after partial removal of headless compression screws, the patient reported increasing pain in her left elbow joint again. The posteroanterior (PA) radiograph showed distinctive posttraumatic OA of the capitellum without any signs of degenerative changes of radial head (Fig. **4a**). According to these findings, the capitellar hemiarthroplasty with capitellar component of the LRE**^TM^** system was primarily detected through a dorsal incision. In contrast to the radiograph, intraoperatively there was additionally a deep circular cartilage defect of radial head due to the formerly backing out of a headless compression screw (Fig. **4a**). Hence, the uncemented radiocapitellar replacement using both components of the LRE**^TM^** system was performed (Fig. **1b**). Postoperative radiographs showed correct alignment of implant without any signs of overstuffing (Fig. **4b**). After surgery, the left arm was immobilized in a plaster splint for two weeks. Then, the elbow's motion was freed, and strengthening was started after the sixth postoperative week.

**Fig. 5:**
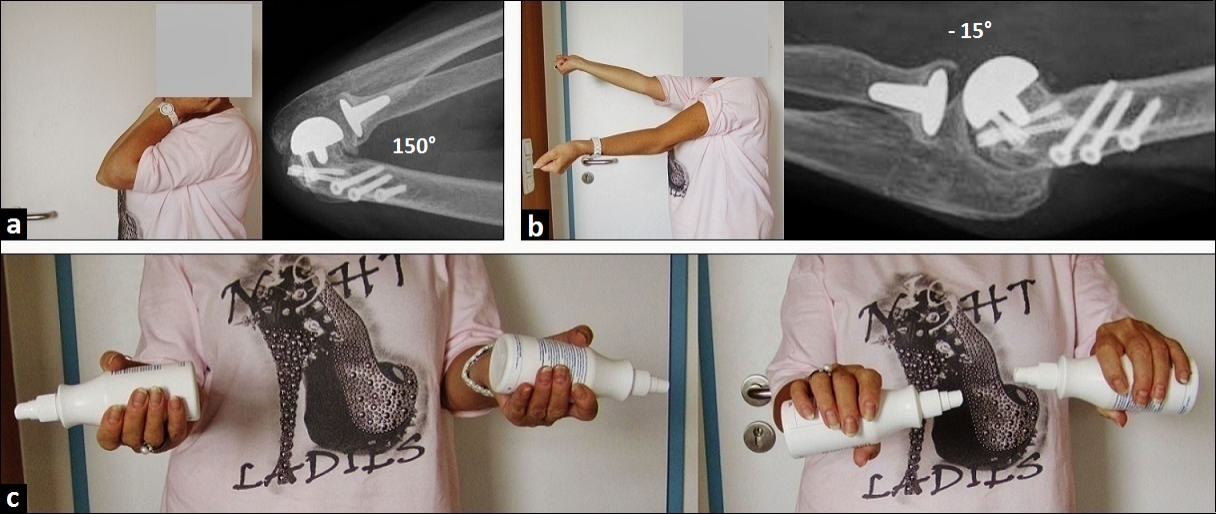
**(Case Presentation): (a)** Clinical photograph and lateral radiograph demonstrating elbow flexion of 150° of both arms, and there is no impingement between both prosthetic components with terminal range of motion. **(b)** Clinical photograph and lateral radiograph of elbow extension demonstrating a deficit of 15° to contralateral, and there is no impingement between both prosthetic components with terminal range of motion. **(c)** Clinical photographs showing each 90° supination and pronation of both forearms.

 At the 2-year follow-up after unicompartmental radiocapitellar replacement; radiographically, there was no change in the position of LRE**^TM^** implant, and with no signs of loosening, subsidence, or overstuffing. Elbow flexion was with 150° equal to contralateral, and the lateral radiograph with terminal range of motion revealed no impingement between both prosthetic components (Fig. **5a**). Elbow extension showed unchanged a deficit of 15° compared to the unaffected right elbow, and the lateral radiograph with terminal range of motion revealed no impingement between both prosthetic components as well (Fig. **5b**) Supination with 90° and pronation with 90° showed no functional deficits compared to the unaffected right forearm (Fig. **5c**). The elbow performance improved from 64 (fair) before LRE**^TM^** arthroplasty to 92 (excellent) in the Mayo Elbow Performance score (MEPS, 0 - 100 points). The patient reported that she would have the same procedure again.

## DISCUSSION

 Although not a weight-bearing joint, the elbow is subjected to considerable forces whose resultant can reach three times the weight of the body at the ulnohumeral and radiocapitellar joints during heavy labour [9]. The mechanism of injury in CSFs is fall on outstreched arm, and often direct axial compression of radial head with capitellum is responsible for fracture pattern [10]. When axial loading is associated with varus stress, the trochlea can be fractured alone, and it is often associated with elbow dislocation [4, 11].

 CSFs are usually associated in up to 40% of cases with lateral collateral ligament injuries, and in up to 30% of cases with radial head fractures [1, 12, 13]. The incidence is higher among women because of their high rate of osteoporosis and carrying angle difference than men [14, 15]. In addition to trauma with or without resulting malunion, unicompartmental radiocapitellar OA may occur by rheumatoid arthritis, osteochondritis dissecans, crystal-induced arthropathies such as gout, sequelae of septic arthritis, and avascular osteonecrosis (Morbus Panner). For the development of degenerative or posttraumatic changes in the elbow, Goodfellow and Bullough [16] first described in 1967 that the articular cartilage with ageing in radiocapitellar joint is much more vulnerable than in ulnohumeral joint, and it can be caused by the specific geometry of elbow joint which is anatomically determined for load transmission.

 ORIF of CSF provides anatomical reduction, stability and early mobilization, and has become the preferred surgical treatment. Closed reduction, immobilization or fragment excision are generally associated with poor outcomes [12, 15]. For ORIF, the use of headless compression screws has proven to be a suitable and reliable option [10, 13, 17]. However, intraarticular migration of a headless compression screw despite complete union of fracture after anatomical reduction, such as in our case, can be a concern. It has also been observed in the wrist in 14% of patients who underwent carpal fusions [18], and can lead in single case to a total wrist arthroplasty [19].

 Generally, treatment options of unicompartmental radiocapitellar OA are excisional procedures or joint replacement. However, capitellar excision creates instability in coronal plane when medial structures are disrupted and poor clinical outcomes were reported in more than 50% of patients [1, 20]. Radial head resection increases the mechanical stresses on the ulnar compartment, worsens the valgus, and carries a risk of proximal radial migration with ulnar variance alteration at the wrist. Isolated radial head resection is therefore controversial [21].

 Resurfacing unicompartmental radiocapitellar replacement offers a new and fascinating option for the treatment of osteoarthritis and rheumatoid arthritis when the lateral compartment is prevalently involved [22, 23]. The aim of unicompartmental radiocapitellar replacement is to obtain stability in elbow joint by avoiding cubitus valgus with subsequent instability of the distal radioulnar joint, and it does not alter the unaffected ulnohumeral joint. The goal of the LRE**^TM^** system is that the radial head is not excised, and so will receive the anatomical length of the overall radius articulating with the capitellum; and additionally, the annular ligament remains preserved. However, in the literature only three publications could be found in which short-term results of resurfing radiocapitellar replacement or capitellar hemiarthroplasty using the LRE**^TM^** system with a total of 36 evaluated patients (averaged 53.3 years old) have been described [6-8]. Twenty-nine patients (80.6%) rated their outcome in MEPS according to Morrey and Adams [24] with excellent or good, four patients (11.1%) with fair, and three patients (8.3%) with poor. None of all implants showed loosening radiographically. Complications in detail were one case with deep infection, one case with a triceps muscle dehiscense, one case with neuropathy of ulnar nerve, three cases with joint stiffness, two cases with heterotopic ossifications, and one case with primary faulty positioning of capitellar component. The elbow's extension deficit of 15° that was observed in our case is mostly based on posttraumatic OA before resurfacing unicompartmental radiocapitellar replacement with the LRE**^TM^** system.

When applying the LRE**^TM^** arthroplasty, the main implant- and/or iatrogenic insertion- related concern is probably overstuffing that was found in 25% of cases by Giannicola et al. [7]. This specific complication is also known with the use of radial head replacement and Copeland resurfing humeral head implant [25, 26]. Overstuffing of elbow joint is defined as radiographic widening of the lateral ulnohumeral joint space due to insertion of an radial head and/or radiocapitellar implant that is too thick, and it can clinically lead to loss of flexion, stiffness, pain and capitellar wear. The diagnosis is often difficult to confirm; for that cases, comparing radiographs of the uninjured elbow is likely the best investigation to consider [27]. Removal of implant combined with radial head resection can be considered as salvage option after a failed unicompartmental radiocapitellar replacement using the LRE**^TM^** system.

## LIST OF ABBREVIATIONS

3D: = Three-dimensional
CoCr: = Cobalt-chrome
CSF(s): = Coronal shear fracture(s)
CT: = Computed tomography
HA: = Hydroxyapatite
LRE: = Lateral Resurfacing Elbow
MEPS: = Mayo Elbow Performance score
OA: = Osteoarthritis
ORIF: = Open reduction and internal fixation
PA: = Posteroanterior
PE: = Polyethylene
UHMW: = Ultra high molecular weight
